# Dose Response of Endotoxin on Hepatocyte and Muscle Mitochondrial Respiration In Vitro

**DOI:** 10.1155/2015/353074

**Published:** 2015-01-12

**Authors:** Victor Jeger, Sebastian Brandt, Francesca Porta, Stephan M. Jakob, Jukka Takala, Siamak Djafarzadeh

**Affiliations:** ^1^Department of Intensive Care Medicine, Inselspital, Bern University Hospital and University of Bern, Freiburgstraße 10, 3010 Bern, Switzerland; ^2^Graduate School for Cellular and Biomedical Sciences, University of Bern, Switzerland; ^3^Department of Anesthesiology and Pain Therapy, Inselspital, Bern University Hospital and University of Bern, 3010 Bern, Switzerland

## Abstract

*Introduction.* Results on mitochondrial dysfunction in sepsis are controversial. We aimed to assess effects of LPS at wide dose and time ranges on hepatocytes and isolated skeletal muscle mitochondria.* Methods.* Human hepatocellular carcinoma cells (HepG2) were exposed to placebo or LPS (0.1, 1, and 10 *μ*g/mL) for 4, 8, 16, and 24 hours and primary human hepatocytes to 1 *μ*g/mL LPS or placebo (4, 8, and 16 hours). Mitochondria from porcine skeletal muscle samples were exposed to increasing doses of LPS (0.1–100 *μ*g/mg) for 2 and 4 hours. Respiration rates of intact and permeabilized cells and isolated mitochondria were measured by high-resolution respirometry. *Results.* In HepG2 cells, LPS reduced mitochondrial membrane potential and cellular ATP content but did not modify basal respiration. Stimulated complex II respiration was reduced time-dependently using 1 *μ*g/mL LPS. In primary human hepatocytes, stimulated mitochondrial complex II respiration was reduced time-dependently using 1 *μ*g/mL LPS. In isolated porcine skeletal muscle mitochondria, stimulated respiration decreased at high doses (50 and 100 *μ*g/mL LPS). *Conclusion.* LPS reduced cellular ATP content of HepG2 cells, most likely as a result of the induced decrease in membrane potential. LPS decreased cellular and isolated mitochondrial respiration in a time-dependent, dose-dependent and complex-dependent manner.

## 1. Introduction

Endotoxin or lipopolysaccharide (LPS) from the outer membrane of Gram-negative bacteria is a trigger of the systemic inflammatory response in sepsis [1]. Mitochondrial dysfunction has been postulated as a mechanism of cell injury and reduced cellular ATP levels in sepsis [2–5]. Sepsis-induced alterations in mitochondrial respiration may account for the lactate production, intracellular acidosis, generation of toxic radicals, and release of cytochrome c from damaged mitochondria. A combination of these factors may contribute to multiorgan failure syndrome in sepsis [6–8]. The effects of LPS on mitochondrial function and, specifically, on mitochondrial respiration are controversial.

LPS triggers macrophages via cell surface receptors (mainly TLR-4) to produce powerful inflammatory mediators including tumor necrosis factor-alpha (TNF-*α*), interleukin-1*α* (IL-1*α*), IL-1*β*, and IL-6. The increased production of these mediators is believed to be the direct cause of LPS toxicity [1, 9–11]. Additionally, many studies demonstrated uptake of LPS by cells [12–16]. Recently, LPS was proposed to act intracellularly, independent of Toll-like receptor 4 [17, 18]. A study on rat cultured hepatocytes demonstrated that LPS binds to the cell membrane and is found afterwards in dispersed localization in the cytoplasm, reaching mitochondria [19]. Consecutively activated caspases might also interact with mitochondria [20, 21]. In hepatocytes, caspase 1 activation reduced mitochondrial respiration and ROS production by increasing mitochondrial autophagy [22].

Research on the effects of LPS on mitochondria has been confounded by the wide range of doses and exposure times. This has likely contributed to the heterogeneous and sometimes contradictory results [23]. Therefore, the aim of the present study was to explore the dose response of LPS on cellular respiration of cultured liver cells and isolated muscle mitochondrial respiration. Hepatocytes were used because (1) liver failure is common in sepsis and (2) intra-abdominal infections are among the main causes of severe sepsis and septic shock [24]; in these circumstances the liver is the first organ to be confronted with (high amounts of) LPS. Muscle mitochondria were used because decreased muscle mitochondrial enzyme activity and ATP content have been reported in sepsis [25, 26]. We hypothesized that endotoxin alters mitochondrial respiration in a dose-dependent way and differently in liver compared to skeletal muscle tissue.

## 2. Materials and Methods

### 2.1. Chemicals and Reagents

LPS (TLR4 agonist, ultrapure* E. coli* K12 lipopolysaccharide) was purchased from InvivoGen (San Diego, CA, USA). All the reagents for cellular respiration were obtained from Sigma-Aldrich (Buchs, Switzerland).

### 2.2. Hepatoma Cell Line (HepG2) and Primary Human Hepatocytes

#### 2.2.1. Cell Culture

Human hepatocellular carcinoma cell line (HepG2, Deutsche Sammlung von Mikroorganismen und Zellkulturen (DSMZ), Braunschweig, Germany, N°ACC 180) was cultured in 25 cm^2^ flasks (for respiration assays) or 96-well plates (for determination of cellular ATP content and mitochondrial membrane potential) in RPMI 1640 culture medium containing 10% heat-inactivated fetal calf serum (FCS), 1 mM sodium pyruvate, 0.1 mM nonessential amino acids, 2 mM L-glutamine, 100 units/mL penicillin, and 100 mg/mL streptomycin at 37°C in a humid atmosphere (5% CO_2_, 95% air). Cells were passaged upon reaching confluence.

The primary human hepatocytes were kindly provided by D. Stroka (University of Bern). Primary human hepatocytes were isolated from wedges of resected liver tissues taken from patients undergoing liver surgery. Written informed consent was obtained prior to surgery in compliance with the local ethical committee [27]. Cultures from 8 donors were prepared and cultured according to Rencurel et al. [28]. Additional primary human hepatocytes (5 donors) were purchased from ScienCell Research Laboratories (Carlsbad, CA, USA) and incubated with LPS and cell viability and respiration rates of intact cells were analyzed.

#### 2.2.2. Experimental Protocol

Quiescent cells were obtained by total deprivation of FCS for 14 to 16 hours before the experiments. All experiments were performed when cells reached 90–95% confluency. On the day of experiment, HepG2 was exposed to placebo or LPS (0.1, 1, 10 *μ*g/mL) for 4, 8, 16, and 24 hours. Primary human hepatocytes were exposed to placebo or 1 *μ*g/mL LPS for 4, 8, and 16 hours. At the end of incubation times, cells were removed by trypsinization, permeabilized, and used for mitochondrial respiration. Due to the limited amount of primary human hepatocytes, only 4, 8, and 16 hours of LPS exposure were explored in these cell types.

#### 2.2.3. Determination of Mitochondrial Respiration in Digitonin-Permeabilized Cells

Respiration was determined as described before [29]. After incubation, cells were trypsinized and resuspended in RPMI-1640 with 10% FBS and then centrifuged for 5 min (350 g). Cell number and viability (using the trypan blue method of dead-cell staining) were determined by the Countess automated cell counter (Invitrogen, Eugene, OR, USA). Cells were resuspended in the respiration buffer (110 mM sucrose, 0.5 mM EGTA, 3.0 mM MgCl2, 80 mM KCl, 60 mM K-lactobionate, 10 mM KH2PO4, 20 mM taurine, 20 mM hepes, 1.0 g/L BSA, and pH 7.1.) at a standard concentration of 1 × 10^6^ cell/mL, and respiration rates were measured at 37°C in in 2 mL glass chambers using the high-resolution oxygraph (Oxygraph-2k, Oroboros Instruments, Innsbruck, Austria). Mitochondrial complex specific respiration rates were assessed by a standard titration protocol: first cells were permeabilized with digitonin (8.1 *μ*M) for 5 min. Afterwards, glutamate (10 mM) and malate (5 mM), which provide nicotinamide adenine dinucleotide (NADH) to the respiratory chain, were added (complex I activation), followed by addition of ADP (0.25 mM). After a stable signal was reached and marked, rotenone (0.5 *μ*M) was added to inhibit complex I and then complex II-dependent respiration was stimulated by adding succinate (10 mM), which provides flavin adenine dinucleotide (FADH) to the respiratory chain (complex II activation). Afterwards, complex III was inhibited by antimycin A (0.5 *μ*M), and complex IV-dependent respiration was measured by adding ascorbate (4 mM) and N,N,N′,N′-tetramethyl-p-phenylenediamine (TMPD, 0.5 mM). Since TMPD exhibits a wide range of autooxidation in the buffer, respiration was finally inhibited with sodium azide (5 mM), and the difference between oxygen consumption before and after addition of sodium azide was interpreted as the real complex IV respiration.

#### 2.2.4. Digitonin Titration

To confirm that mitochondrial outer membrane integrity was not compromised in permeabilized cells, we performed digitonin titration to determine the optimal concentration for permeabilization of HepG2 cells and primary human hepatocytes. For these experiments, cells were suspended in respiration buffer (1 × 10^6^ cells/mL) and digitonin concentration was titrated in intact cells by respirometry in the presence of 0.5 mM rotenone (to inhibit endogenous respiration of intact cells), 10 mM succinate, and 1 mM ADP. Respiration rates were measured at baseline and after 2–5 min after each titration. Respiration of intact HepG2 cells and primary hepatocytes was not stimulated in the presence of rotenone, succinate, and ADP. However subsequent stepwise digitonin titration yielded gradual permeabilization of plasma membranes, shown by the increase of respiration up to full permeabilization. Permeabilization at a digitonin concentration of 8–10 *μ*M was optimum for ADP-stimulated respiration.

#### 2.2.5. Coupled and Uncoupled Respiration of Intact Cells: Cellular Respiration

After incubation with LPS (HepG2: 1 *μ*g/mL, 24 hours; primary human hepatocytes: 1 *μ*g/mL, 24 hours), cells were trypsinised and resuspended in RPMI-1640 with 10% FBS and then centrifuged for 5 min (350 g). Cells were resuspended in the respiration buffer (110 mM sucrose, 0.5 mM EGTA, 3.0 mM MgCl2, 80 mM KCl, 60 mM K-lactobionate, 10 mM KH2PO4, 20 mM taurine, 20 mM hepes, 1.0 g/L BSA, and pH 7.1) at a concentration of 1 × 10^6^ cells/mL. Respiration rates were measured at 37°C using a high-resolution oxygraph (Oxygraph-2k, Oroboros Instruments, Innsbruck, Austria). Basal coupled endogenous respiration of intact cells (oxygen consumption without the addition of exogenous substrate) was measured and recorded using the linear rate of oxygen consumption. Afterwards, oligomycin (an inhibitor of ATP synthase) (0.4 *μ*g/mL) was added and the nonphosphorylating respiration rate was measured (oligomycin-insensitive respiration). Afterwards the chemical uncoupler FCCP (carbonyl cyanide p-trifluoromethoxyphenylhydrazone) was sequentially added at different concentrations (0.1 to 0.5 *μ*M) and maximal uncoupled respiration was recorded. Thereafter, respiration was inhibited with the addition of rotenone (0.5 *μ*M) and antimycin A (0.5 *μ*M) and the remaining background respiration was subtracted from all results. The uncoupled respiratory control ratio (uRCR) was calculated as the ratio between the oxygen consumption rate in the presence of FCCP and the rate in the presence of oligomycin. The oligomycin-sensitive respiration (ATP turnover) was calculated by subtracting the oligomycin-insensitive respiration from basal endogenous respiration. The coupling efficiency corresponded to the ratio of oligomycin-sensitive respiration rates by basal respiration rates.

#### 2.2.6. Measurement of Mitochondrial Membrane Potential in Intact Cells

The mitochondrial electrochemical potential gradient (Δ*ψ*m) in intact cells was measured using the cationic dye 5,5′,6′,6′-tetrachloro-1,1′,3,3′-tetraethylbenzimidazolo-carbocyanine iodide (JC-1). JC-1 is a mitochondrial sensor which aggregates in polarized mitochondria, where it forms red fluorescent aggregates. Dissipation of the mitochondrial membrane potential prevents the accumulation of the JC-1 dye in the mitochondria, and the dye is dispersed throughout the entire cell, leading to a shift from red (JC-1 aggregates) to green fluorescence (JC-1 monomers). Thus the loss of JC-1 aggregates directly correlates with changes in Δ*ψ*m. Briefly, for these experiments, the cells were grown in 96-well plates and treated with LPS for 24 hours (1 *μ*g/mL) and the mitochondrial membrane potential was measured using the JC-1 mitochondria staining kit for mitochondrial potential change detection (Sigma, Switzerland) according to the manufacturer's instructions. Δ*ψ*m was measured immediately by fluorimetry. For JC-1 monomers, the fluorimeter was set at a 490 nm excitation wavelength and 530 nm emission wavelength and fluorescence was measured. For JC-1 aggregates, the fluorimeter was set at a 525 nm excitation wavelength and 590 nm emission wavelength and fluorescence was measured. Afterwards, the Δ*ψ*m (590/530 nm fluorescence ratio) was calculated. Valinomycin (2.5 *μ*g/mL) was used as a positive control to depolarize the mitochondrial membrane potential.

#### 2.2.7. Cytochrome c Test

In some additional experiments, to evaluate the quality of the mitochondria, cytochrome c test was performed to verify outer mitochondrial membrane integrity. For these experiments, HepG2 and primary human hepatocytes were incubated with LPS and permeabilized with digitonin (8.1 *μ*M) and mitochondrial outer membrane integrity was tested by measurement of mitochondrial respiration after the subsequent additions of glutamate (10 mM) and malate (5 mM) and/or succinate (10 mM), ADP (0.25 mM), and cytochrome c (10 *μ*M) followed by the addition of oligomycin (0.4 *μ*g/mL) to mimic state 4.

#### 2.2.8. Measurement of the HepG2 Cells ATP Content

For these experiments, the cells were grown in 96-well plates and treated with LPS for 24 hours (1 *μ*g/mL). After treatment, cells were lysed and exposed to the ATP substrate solution using the Luminescent ATP Detection Assay Kit (Abcam, Cambridge, USA) according to the manufacturer's instructions and signal was measured on a luminescent counter. The assay kit is a cellular ATP monitoring system based on firefly (Photinus pyralis) luciferase and is based on the production of light caused by the reaction of ATP with added luciferase and D-luciferin. The emitted light is proportional to the ATP concentration inside the cell. Valinomycin (2.5 *μ*g/mL) was added to assess the effect of depolarized mitochondrial membrane potential on ATP content.

#### 2.2.9. Measurement of Release of Reactive Oxygen Species (ROS) and Reactive Nitrogen Species (RNS)

Cells were grown in 96-well plates and treated with LPS for 24 hours (1 *μ*g/mL). Total free radicals (ROS and RNS) levels were measured in the supernatant using the OxiSelect In Vitro ROS/RNS Assay Kit (Cell Biolabs Inc., San Diego, CA, USA). ROS and RNS species react with DCFH to the fluorescent 2′,7′-dichlorodihydrofluorescein (DCF). Its fluorescence intensity is proportional to the total ROS and RNS levels within the sample and was measured by fluorimetry.

### 2.3. Skeletal Muscle Mitochondria

#### 2.3.1. Mitochondrial Isolation

Quadriceps muscle biopsy was taken from 7 anaesthetized pigs, from which mitochondria were isolated by differential centrifugation as described by Porta et al. [30]. Protein concentration was determined using the Quant-itTM Protein Assay kit and read in QubitTM fluorometer (Invitrogen, Basel, Switzerland). Pigs were used afterwards for another experiment. The study was performed in accordance with the National Institutes of Health guidelines for the care and use of experimental animals and with the approval of the Animal Care Committee of the Canton Bern, Switzerland.

#### 2.3.2. Experimental Protocol

The protocol started immediately after isolation of the mitochondria, and determination of respiration was finished within 6 hours in all groups. Mitochondria from each biopsy sample were divided into 12 groups and incubated on ice with vehicle or 0.1, 1, 10, 50, and 100 *μ*g LPS/mg mitochondrial protein both for 2 hours and for 4 hours. The different concentrations at 2 or 4 hours were assessed in a randomized fashion. The data were compared with its time-matched controls to prevent a time effect due to a longer delay after isolation.

#### 2.3.3. Determination of Mitochondrial Respiration in Isolated Mitochondria

Mitochondrial respiration was performed as described before [31]. Respiratory rates were determined at a final mitochondrial protein concentration of 0.4 mg/mL in the above-described respiration buffer. Respiration was measured at 37°C in 2 mL glass chambers using the high-resolution oxygraph (Oxygraph-2k, Oroboros Instruments, Innsbruck, Austria). Each complex was assessed with a separate mitochondrial sample. Maximal oxidative capacities (state 3) were determined in the presence of oxygen content of room air (21%). For complex I-dependent respiration, substrates were glutamate (10 mM) + malate (5 mM) followed by ADP (0.025 mM). For measurement of complex II-dependent respiration, succinate (10 mM) and ADP (0.025 mM) were added. The coupling of phosphorylation to oxidation was determined by calculating the respiratory control ratio (RCR) as the ratio between ADP-stimulated respiration (state 3) and respiration after ADP depletion (state 4). Complex IV-dependent respiration was measured by adding ascorbate (4 mM), N,N,N′,N′-tetramethyl-p-phenylenediamine (TMPD, 0.5 mM) and ADP (0.025 mM). Since TMPD exhibits a wide range of autooxidation in the buffer, respiration was finally inhibited with sodium azide (5 mM), and the difference between the oxygen consumption before and after the addition of sodium azide was interpreted as the real complex IV respiration. Respiration rates were calculated as the time derivative of oxygen concentration measured in the closed respirometer and expressed per million viable cells. The amplified signal was recorded in a computer with online display of the calibrated oxygen concentration and oxygen flux (DatLab software for data acquisition and analysis; Oroboros Instruments, Innsbruck, Austria). Oxygen consumption is expressed as pmol O_2_/s/mg of mitochondrial protein.

### 2.4. Statistics

Statistical analysis was performed with the SPSS software package (SPSS 15.0, SPSS Inc., Chicago, IL) and Prism 6 (GraphPad Software, La Jolla, CA). Mitochondrial respiration of each complex at different doses of LPS was compared at each time point using the nonparametric Friedman test followed by the Dunn's test for multiple comparisons. If significant, the Wilcoxon test was used to compare this dose with its control. For primary human hepatocytes, the Wilcoxon test was used as only one dose of LPS was explored, due to the limited availability of primary human hepatocytes. For unpaired data (ATP content, mitochondrial membrane potential) the Mann-Whitney test was used. Data are shown as median and interquartile range (IQR).

## 3. Results

### 3.1. Hepatoma Cell Line (HepG2)

**Figure 1:**
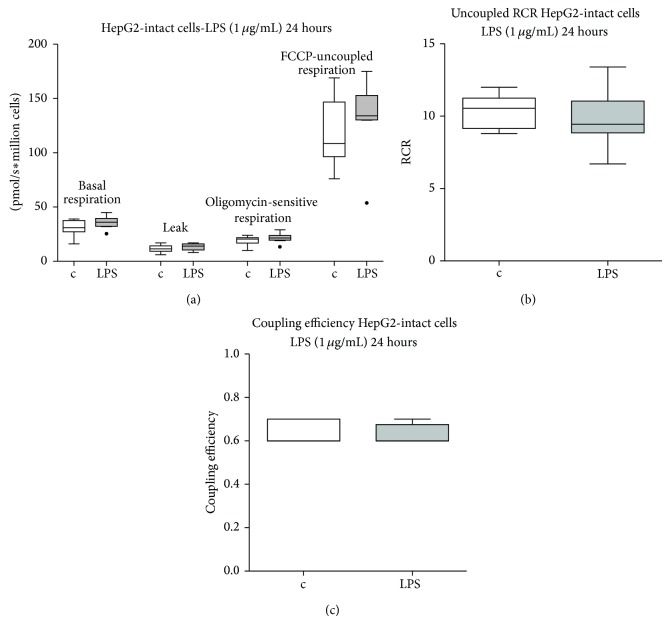
Basal respiration of intact cells. HepG2 cells' basal oxygen consumption, in the presence of oligomycin (leak respiration), and FCCP, respectively, in LPS treated and control cells ((a); *n* = 8 each). The uncoupled respiratory control ratio (uRCR) was calculated as the ratio between the oxygen consumption rate in the presence of FCCP and the rate in the presence of oligomycin (b) and the coupling efficiency was calculated as the ratio between oligomycin-sensitive and basal respiration rates (c). Data represented as box and whiskers (Tukey). Statistical significance between samples was assessed using a Wilcoxon test. c: control; LPS: 1 μg/mL LPS for 24 hours.

For the measurement of cellular respiration of intact cells, cells were exposed to LPS and respiration rates were measured in the absence of exogenous substrates and ADP. LPS at 1 *μ*g/mL for 24 hours did not affect basal endogenous cellular respiration of intact cells, oligomycin-insensitive (nonphosphorylating respiration), oligomycin-sensitive (ATP turnover), and FCCP-uncoupled maximal respiration rates (Figure 1(a)). Neither uncoupled respiratory control ratio (the ratio between FCCP and oligomycin-insensitive respiration rates) nor coupling efficiency (the ratio between oligomycin-sensitive and basal respiration rates) were affected (Figure 1(b)). LPS did not affect cellular viability (median, IQR: 89% (85%–90%) live cells in controls versus 88% (86%–90%), in stimulated cells).

**Figure 2:**
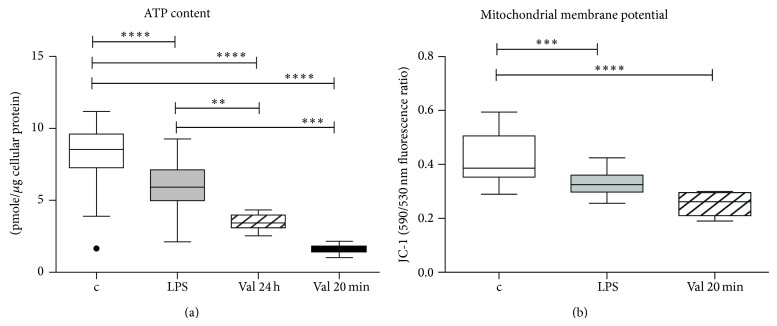
ATP content and mitochondrial membrane potential of intact cells. (a) Cellular ATP content of HepG2 was determined after 24 hours exposure with 1 μg/mL LPS (*n* = 90), control (*n* = 102), valinomycin (2.5 μg/mL) for 24 h (*n* = 12), and valinomycin for 20 minutes (*n* = 12). Kruskal-Wallis *P* < 0.0001. (b) Mitochondrial membrane potential measured by the changes in the 590/530 JC-1 emitted fluorescence. Control: *n* = 30, LPS: *n* = 24. Valinomycin (2.5 μg/mL; incubation for 20 minutes) served as a positive control, to depolarize mitochondrial membrane potential (*n* = 6). Data are represented as box and whiskers (Tukey). Groups were compared using Mann-Whitney *U* test (two groups) or Kruskal-Wallis test followed by Dunn's correction of multiple comparisons (three groups). (c: control; LPS: lipopolysaccharide; val: valinomycin.)

**Figure 3:**
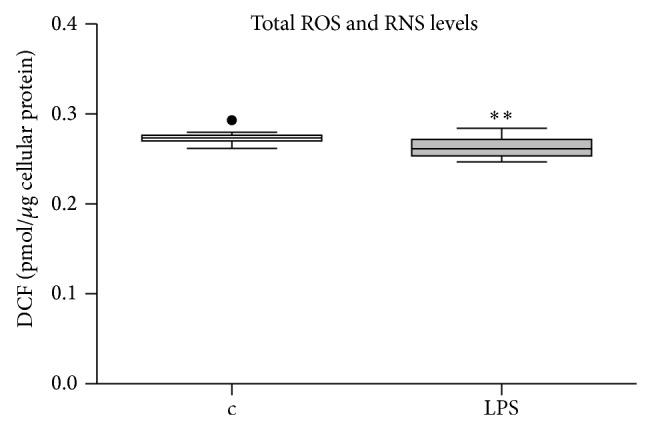
Measurements of total free radical ROS and RNS levels in supernatants of intact cells. Total reactive oxygen species (ROS) and reactive nitrogen species (RNS) were determined after reaction with DCFH to DCF (2′,7′-dichlorodihydrofluorescein) in supernatants of LPS treated and control cells (*N* = 16 each). The fluorescence of DCF is proportional to the content of ROS and RNS in the cell culture medium. DCF was expressed per pmol DCF/μg cellular protein. Data are represented as box and whiskers (Tukey). Groups were compared using Mann-Whitney *U* test. ^∗∗^ *P* = 0.0052.

LPS at 1 *μ*g/mL for 24 hours decreased cellular ATP content and mitochondrial membrane potential in intact cells (Figure 2). Valinomycin, which was used as a positive control and is known to depolarize mitochondrial membrane potential, did induce a significant decrease in mitochondrial membrane potential and cellular ATP content (Figure 2). LPS did not alter reactive oxygen species (ROS) and reactive nitrogen species (RNS) concentration in the cell culture medium (Figure 3).

**Figure 4:**
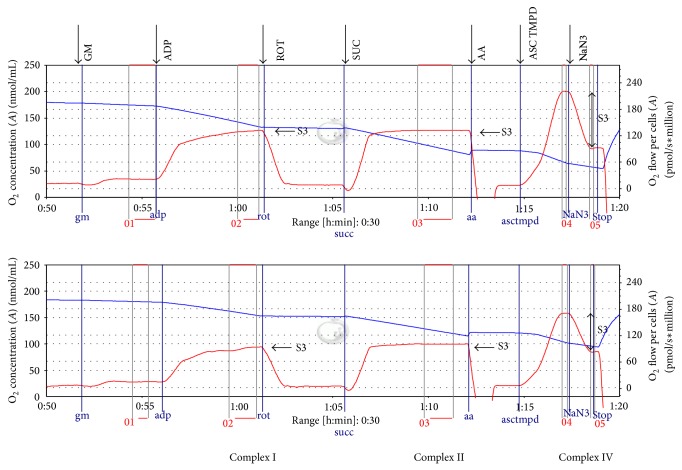
Tracings from the OROBOROS-high-resolution respirometry of permeabilized cells. Legend: the upper tracing represents a cell sample incubated with placebo (control) for 24 hours; the lower tracing represents a cell sample at the same passage number incubated with 1 μg/mL LPS for 24 hours. The experiments were recorded simultaneously. The blue line represents oxygen concentration; the red line represents the oxygen flow (slope of oxgygen concentration). GM: Glutamate + malate, ADP: adenosine diphosphate, ROT: rotenone, SUC: succinate, AA: antimycin A, ASC TMPD: ascorbate + N,N,N′,N′-tetramethyl-p-phenylenediamine, NaN_3_: sodium azide, and S3: state 3. State 4 cannot be measured due to the saturating concentration of ADP during the experiment.

Representative respiration rates of permeabilized HepG2 cells using high-resolution respirometry are shown in Figure 4 (*n* = 10 for each experiment). 24 hours of incubation with LPS (1 *μ*g/mL) induced a significant reduction in maximal complex II-dependent and IV-dependent respiration of HepG2 cells but did not affect cell viability (Table 1). 0.1 and 10 *μ*g LPS/mL as well as 4, 8, and 16 hours of incubation induced no significant changes.

### 3.2. Isolated Primary Human Hepatocytes

**Figure 5:**
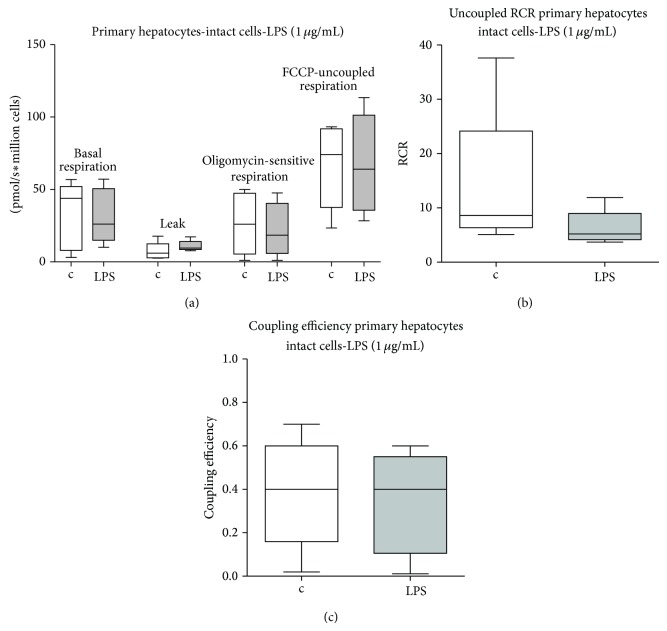
Basal respiration of intact cells. Primary human hepatocytes' basal oxygen consumption, in the presence of oligomycin (leak respiration), and FCCP, respectively, in LPS treated and control cells ((a); *n* = 5 each). The uncoupled respiratory control ratio (uRCR) was calculated as the ratio between the oxygen consumption rate in the presence of FCCP and the rate in the presence of oligomycin (b) and the coupling efficiency was calculated as the ratio between oligomycin-sensitive and basal respiration rates (c). Data represented as box and whiskers. Statistical significance between samples was assessed using a Wilcoxon test. c: control; lps: 1 μg/mL LPS for 8 hours.

Basal respiration rates were determined in intact primary human hepatocytes in the absence of exogenous substrates (Figure 5). There were no significant differences after exposure to 1 *μ*g/mL LPS (8 hours incubation).

1 *μ*g/mL LPS reduced complex II-dependent but not complex I-dependent or complex IV-dependent respiration of isolated and permeabilized human hepatocytes after 8 hours incubation (*P* = 0.016; Table 2). LPS did not affect viability of isolated primary human hepatocytes (Table 2).

#### 3.2.1. Cytochrome c

Cytochrome c did not enhance respiration of the HepG2 or primary human hepatocytes treated with placebo or LPS indicating that there was no loss of cytochrome c from mitochondrial outer membrane and mitochondrial integrity was preserved (data not shown).

### 3.3. Isolated Mitochondria

Control mitochondria kept on ice for 4 hours were significantly lower compared to the same mitochondria analyzed 2 hours after isolation. To prevent the time effect, each exposed sample of mitochondria was compared to its time-matched control sample. Two-hour incubation with 50 and 100 *μ*g LPS/mg mitochondrial protein reduced complex I state 3, complex I state 4, and complex II state 3 dependent mitochondrial oxygen consumption (Table 3).

## 4. Discussion

LPS did not impair basal respiration in intact HepG2 cells. Despite maintained oxygen consumption, cellular ATP content was reduced, likely as a result of the induced reduction of the mitochondrial membrane potential. The effects were similar, albeit less strong, than those of valinomycin, an ionophore capable of depolarizing mitochondria [32]. Others confirmed the mitochondrial potential lowering characteristics of LPS in hepatocytes [33–35]. LPS may cause recruitment of an uncoupling protein (UCP2) both in vivo and in vitro and thereby decrease the mitochondrial membrane potential [34]. LPS can also induce ROS production and loss of mitochondrial membrane potential by TNF-*α* and IFN-*γ* upregulation via iNOS induction and NO production [35].

Mitochondrial oxygen consumption was only affected by LPS when respiration was stimulated with complex specific substrates (e.g., succinate for complex II). In primary human hepatocytes LPS induced a reduction in complex II-dependent respiration rates. Due to the limited availability of primary human hepatocytes, we evaluated further effects of LPS in HepG2 cells, a human hepatoma cell line. Also in HepG2 cells, LPS reduced stimulated respiration. Finally, maximal respiration was also impaired in isolated skeletal muscle mitochondria.

Our data confirm the findings of a previous review on effects of sepsis on mitochondrial functions and may explain the heterogenous results in the literature: LPS effects seem to be time, dose, tissue, and method dependent. We found impaired respiration after 8 hrs (human hepatocytes) or only after 24 hrs of LPS incubation (HepG2 cells) and at 1 *μ*g LPS/mL (hepatocytes) or at only 50 and 100 *μ*g LPS/mL (isolated skeletal mitochondria). Furthermore, mitochondrial complexes I, II, or IV could be implicated alone or in various combinations. Finally, we demonstrate that immediate processing of tissue samples is crucial for detection of LPS effects. This is especially important when time-consuming procedures like differential centrifugation are involved.

Transient effects of LPS on primary hepatocytes' mitochondrial respiration have been reported previously [33]. In a former experimental series, we have also reported transient effects of TLR-3 stimulation on HepG2 and postulated mitochondrial biogenesis to be involved in mitochondrial recovery [29]. New insights to this phenomenon come from a recently published article: it was proposed that, in reaction to LPS or sepsis, mitochondrial oxygen consumption is reduced as a protective mechanism to prevent cell death [33]. During recovery, the cell may get rid of dysfunctional mitochondria by autophagy and seems to activate mitochondrial biogenesis via TLR-9 stimulation in autophagosomes, which could lead finally to a restored function of mitochondria [33].

LPS is recognized by TLR4, which is present not only on the surface of immune cells but also on the surface of hepatocytes [1, 36, 37], including HepG2 cells [38, 39]. However, their density on the cell surface may vary. The binding of LPS on TLR4 activates complex intracellular pathways leading to the activation of transcription factors like NF*κ*B, which then results in the release of mediators of the immune system [40]. TLR1/2/4 signaling drives mitochondria in macrophages to increase reactive oxygen species (ROS) production in response to bacteria [41]. West et al. suggested a direct signaling pathway that links TLR's with mitochondria [41]. In HepG2 cells in the present study ROS and RNS levels were not increased after 24 h exposure to LPS. It has been described that the increase in ROS is transient [42], and we may have missed the peak in our study.

In addition to a TLR4 mediated pathway, a mechanism of LPS endocytosis is postulated and it has been shown that LPS can reach mitochondria directly [12–16, 19]. Therefore, we evaluated the effect of increasing LPS doses at different exposure times on isolated skeletal muscle mitochondria from healthy pigs. We observed impaired mitochondrial respiration only at high LPS doses. We did not observe any effect on respiratory control ratio (RCR) of isolated muscle mitochondria because of the simultaneous decrease of state 3 and state 4. However, others found a reduced RCR after exposure of isolated mitochondria to LPS, either by a decrease in state 3 or by an increase in state 4 [43, 44]. The mechanism of a direct LPS effect on mitochondria is not well understood. Recently, TLR-4 independent activation of caspase 11 by intracellular LPS was proposed, which could lead to either IL-1 activation or cell death [17, 18]. The intermediates or receptors for intracellular LPS are not known. Furthermore, there are reports about intracellular TLR4 receptors in macrophages and human coronary artery endothelia cells, which react to LPS stimuli [45, 46]. But till now, there is no evidence of any interaction of intracellular TLR4 and mitochondria. Therefore, it is not known how LPS induces isolated mitochondrial dysfunction directly.

In HepG2 and primary human hepatocytes, LPS decreased cellular respiration in the presence of exogenous substrates (permeabilized cells) without impairing basal respiration of intact cells. A limitation of the present study is that most of the cellular experiments were performed in HepG2 cells, due to the limited availability of primary human hepatocytes. HepG2, a human hepatoma cell line, has a different phenotype compared to primary hepatocytes and is thought to be a mature epithelial cell line, which can form structures similar to bile canaliculi [47]. HepG2 has a lower rate of triglycerides secretion than primary hepatocytes and resembles more fasted primary hepatocytes [47]. Furthermore, HepG2 has very low levels of functional cytochrome P450 enzymes compared to primary hepatocytes [48]. However, HepG2 contains a high level of mitochondria and mtDNA and is therefore a good model to investigate mitochondrial toxicity [49]. It should be noted that HepG2 cells as cancer cells have distinct metabolism involving oxidative glutaminolysis (nonexisting in primary noncancerous hepatocytes) and their ability to switch between aerobic glycolysis and and oxidative phosphorylation, thus varying contribution of these two to overall ATP. This is in contrast to primary hepatocytes in which 99% ATP originates from oxidative phosphorylation. Permeabilization may on one hand equalize these distinctions, but not the different enzyme composition pattern of HepG2 cells versus primary hepatocytes. Limitations in the use of primary human hepatocytes are their limited availability, instability in vitro, and substantial variability if derived from different donors [48].

## 5. Conclusion

LPS decreased mitochondrial membrane potential and cellular ATP content in intact HepG2 cells without impairing basal respiration. However, maximal (stimulated) respiration was decreased in the presence of exogenous substrates (permeabilized cells) in a time-dependent and dose-dependent fashion. In primary hepatocytes, LPS decreased as well as cellular respiration in the presence of exogenous substrates (permeabilized cells) without impairing basal respiration.

Our data confirm the heterogeneous reaction of mitochondrial respiration to LPS.

## Figures and Tables

**Table 1:** Effects of LPS exposure on cellular respiration and cell viability of cultured, digitonin-permeabilized human hepatoma cells (HepG2) for various time intervals and at different concentrations. For cellular respiration, all values are expressed as pmol O_2_/(sec ∗ Mill. Cells); ^∗∗^ *P* < 0.01; *n* = 10 each (LPS treated and control cells); data are expressed as median (IQR). Friedman test followed by Dunn's correction of mutliple comparisons was used to detect differences between doses of LPS at each time point. If significant, a Wilcoxon test was used to compare the difference compared to the corresponding control group. Wilcoxon of complex II 24 hours 1 *μ*g LPS/mL versus control: *P* = 0.002; Wilcoxon of complex IV 24 hours 1 *μ*g LPS/mL versus control: *P* = 0.004.

HepG2 permeabilized		Control	0.1 *μ*g LPS/mL	1 *μ*g LPS/mL	10 *μ*g LPS/mL	Friedman *P*
Complex I	4 h	99 (86–112)	107 (88–115)	105 (101–111)	106 (98–119)	0.430
	8 h	90 (78–99)	89 (77–105)	89 (82–96)	88 (76–95)	0.392
	16 h	95 (68–101)	96 (83–106)	95 (84–101)	89 (81–99)	0.472
	24 h	96 (82–110)	105 (92–111)	87 (84–97)	91 (77–110)	0.339

Complex II	4 h	121 (107–126)	115 (108–134)	123 (109–127)	124 (114–130)	0.516
	8 h	126 (109–135)	117 (95–133)	131 (118–145)	125 (99–135)	0.339
	16 h	104 (81–129)	120 (91–132)	115 (95–126)	108 (95–126)	0.669
	24 h	94 (78–111)	94 (84–105)	^**^76** (68–93)**	84 (67–99)	**0.006**

Complex IV	4 h	172 (166–187)	179 (155–210)	170 (166–181)	174 (161–185)	0.669
	8 h	166 (97–200)	144 (118–166)	145 (126–168)	147 (129–173)	0.840
	16 h	139 (90–171)	127 (108–165)	123 (111–156)	122 (109–174)	0.840
	24 h	98 (90–130)	99 (84–118)	^**^81** (63–93)**	98 (89–133)	**0.007**

Viability (%)	8 h	84 (68–91)	87 (72–95)	88 (71–93)	88 (70–92)	
	16 h	95 (93–96)	93 (90–94)	94 (91–95)	94 (90–95)	
	24 h	94 (92–95)	92 (89–93)	94 (93–95)	93 (90–95)	

**Table 2:** Time course of 1 *μ*g/mL LPS on oxygen consumption and cell viability of isolated and permeabilized human hepatocytes. 8 hours of incubation with 1 *μ*g/mL LPS reduces complex II-dependent respiration compared to control (^∗^ *P* = 0.03). For cellular oxygen consumption, all values are expressed as pmol O_2_/(sec ∗ Mill. Cells). A Wilcoxon test for non-parametric paired data was used to compare the control group with the LPS group at each time point. *N* = 8 for 4 and 16 hours, *n* = 7 for 8 hours in LPS treated and control cells.

Primary hepatocytes permeabilized		Control	1 *μ*g LPS/mL	*n*	*P*
Complex I	4 h	252 (91–351)	287 (116–337)	8	0.547
	8 h	248 (93–340)	198 (88–293)	7	0.156
	16 h	191 (112–368)	172 (113–329)	8	0.641

Complex II	4 h	411 (198–474)	342 (201–510)	8	0.844
	8 h	**454 (242–523)**	^*^365** (167–511)**	7	**0.016**
	16 h	317 (239–530)	274 (241–516)	8	0.742

Complex IV	4 h	242 (192–308)	256 (146–346)	8	0.641
	8 h	257 (167–350)	203 (143–383)	7	0.375
	16 h	287 (208–362)	308 (179–388)	8	0.844

Viability (%)	control	98 (93–99)		5	
	4 h		91 (83–95)	5	
	8 h		99 (99-100)	5	
	16 h		97 (93–99)	5	

**Table 3:** Time and dose course of LPS on isolated pig skeletal muscle mitochondria. Data are expressed as median (IQR). All values are in pmol O_2_/(sec ∗ mg mitochondrial protein). *N* = 7 for each experiment in LPS treated and control isolated mitochondria. ^∗^Wilcoxon *P* < 0.05 compared to its time-matched control sample. A Friedman test was used to compare a dose effect at each time point. If significant, a Wilcoxon test was performed to compare the difference to its time-matched control sample (^∗^ *P* < 0.05). Additionally, a Wilcoxon test was performed to detect differences in control samples measured 2 and 4 hours after isolation (^§^ *P* < 0.05).

Isolated mitochondria		Control	0.1 *μ*g LPS/mg	1 *μ*g LPS/mg	10 *μ*g LPS/mg	50 *μ*g LPS/mg	100 *μ*g LPS/mg	Friedman *P*
Complex I state 3	2 h	1676 (1171–1914)	1512 (1093–1527)	1365 (1110–1790)	1396 (992–1591)	^*^1130** (725–1562)**	^*^1035** (788–1443)**	**0.008**
	4 h	**1178 **(572–1588)^§^	1250 (493–1742)	1050 (657–1548)	1207 (407–1814)	1258 (584–1543)	1081 (804–1424)	0.576

Complex I state 4	2 h	454 (306–556)	420 (267–525)	418 (259–558)	419 (198–526)	^*^342** (162–413)**	^*^309** (171–423)**	**0.003**
	4 h	269 (191–445)	309 (175–427)	310 (205–346)	361 (149–448)	334 (204–532)	332 (201–510)	0.846

Complex I RCR	2 h	3.3 (2.7–4.5)	3.6 (2.4–4.3)	3.3 (2.9–5.7)	3.4 (2.5–3.8)	3.4 (1.9–4.6)	4.3 (2.6–5.3)	0.670
	4 h	3.6 (3.0–4.9)	4.1 (2.8–4.2)	4.1 (2.5–4.9)	4.1 (2.3–4.6)	3.9 (2.4–4.6)	3.5 (2.4–5.0)	0.946

Complex II state 3	2 h	1753 (469–2340)	1479 (449–2046)	1042 (476–1671)	1468 (388–2430)	^*^879** (444–1320)**	^*^1070** (414–1217)**	**0.005**
	4 h	**1308 **(402–1444)^§^	1428 (459–1680)	1181 (425–1594)	1359 (326–1495)	958 (386–1019)	754 (479–1097)	0.136

Complex II state 4	2 h	631 (398–882)	576 (363–902)	578 (413–683)	591 (323–866)	627 (300–989)	637 (293–890)	0.600
	4 h	716 (322–888)	732 (375–891)	679 (337–803)	599 (284–1086)	618 (311–923)	595 (323–656)	0.227

Complex II RCR	2 h	2.2 (1.4–2.7)	2.0 (1.2–2.6)	2.0 (1.2–2.6)	2.0 (1.2–2.5)	1.2 (1.1–1.8)	1.4 (1.2–2.1)	0.123
	4 h	1.3 (1.1–2.3)	1.9 (1.1–2.1)	1.3 (1.1–2.3)	1.3 (1.2–1.9)	1.3 (1.2–1.7)	1.4 (1.2–1.9)	0.923

Complex IV state 3	2 h	2338 (684–4335)	1762 (415–4223)	977 (690–2325)	1912 (435–3679)	1890 (379–2722)	1553 (368–2379)	0.084
	4 h	**1488 **(444–1927)^§^	1553 (399–3516)	1356 (399–2320)	1276 (422–3043)	2145 (450–2344)	979 (388–1643)	0.068
